# Histopathological Image Deep Feature Representation for CBIR in Smart PACS

**DOI:** 10.1007/s10278-023-00832-x

**Published:** 2023-06-09

**Authors:** Cristian Tommasino, Francesco Merolla, Cristiano Russo, Stefania Staibano, Antonio Maria Rinaldi

**Affiliations:** 1 Department of Electrical Engineering and Information Technology, University of Napoli Federico II, Via Claudio 21, Naples, 80125 Italy; 2 Department of Medicine and Health Sciences V. Tiberio, University of Molise, Campobasso, 86100 Italy; 3 Department of Advanced Biomedical Sciences, Pathology Section, University of Naples Federico II, Naples, 80131 Italy

**Keywords:** Deep learning, Computational pathology, Content-based image retrieval, PACS

## Abstract

Pathological Anatomy is moving toward computerizing processes mainly due to the extensive digitization of histology slides that resulted in the availability of many Whole Slide Images (WSIs). Their use is essential, especially in cancer diagnosis and research, and raises the pressing need for increasingly influential information archiving and retrieval systems. Picture Archiving and Communication Systems (PACSs) represent an actual possibility to archive and organize this growing amount of data. The design and implementation of a robust and accurate methodology for querying them in the pathology domain using a novel approach are mandatory. In particular, the Content-Based Image Retrieval (CBIR) methodology can be involved in the PACSs using a query-by-example task. In this context, one of many crucial points of CBIR concerns the representation of images as feature vectors, and the accuracy of retrieval mainly depends on feature extraction. Thus, our study explored different representations of WSI patches by features extracted from pre-trained Convolution Neural Networks (CNNs). In order to perform a helpful comparison, we evaluated features extracted from different layers of state-of-the-art CNNs using different dimensionality reduction techniques. Furthermore, we provided a qualitative analysis of obtained results. The evaluation showed encouraging results for our proposed framework.

**Graphical Abstract:**

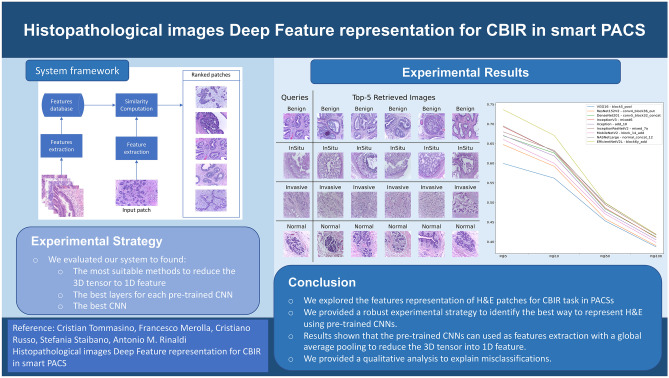

## Introduction

Pathology departments are increasingly using or proposing to use digital pathology technologies for all or some of their diagnostic outputs. Some departments have already moved to a digital workflow, implementing digital scanning technology in routine diagnostics and even including artificial intelligence (AI) technologies for evaluating some specimens (such as prostate biopsies) in everyday work [1]. Whole slide imaging (WSI), whose technology is constantly evolving, may be used to digitally turn a histological tissue section on a glass slide into a high-resolution virtual slide. During the past 20 years, digital scanners, image visualization tools, and algorithms generated from artificial intelligence have increased tremendously. Following the US FDA’s recent authorization of a WSI system for use in primary surgical pathology diagnosis, there are now more chances for widespread adoption and practical application of WSI technology in pathology units [2]. The storage, retrieval, and analysis of biomedical images are essential tools of Picture Archiving and Communication Systems (PACSs) [3, 4]. The same ones are also useful in related contexts such as computational pathology (CPATH) [5], where their diffusion is still limited. A weakness of traditional PACSs concerns the capability to perform a query only employing metadata. In order to allow intelligent multimodality query posing [6], Content-Based Image Retrieval (CBIR) techniques can be used [7, 8]. The feature extraction methods and similarity functions are crucial aspects of a CBIR [9]. Over the years, different approaches for image retrieval have been proposed, also in the computational pathology field using different techniques such as deep learning [10]. Such technologies based on deep convolutional architecture trained on large datasets can learn features with sufficient representational power and generalization to perform semantic visual discrimination tasks [11]. Such a feature is called DeCAF or Deep Feature. Subsequently, many works used CNN as a feature extractor in several applications, including image retrieval [12–16]. Many challenges in CPATH have widely applied deep learning [17]. In fact, as reported in different papers [17–19], the automatic tools that involve deep learning techniques are helpful for pathologists. Moreover, CBIR systems in this domain require different features from others. The main difference regards the kind of available images. In the computational pathology context, we have WSIs, which are gigapixel images composed of thousands of pixels that depict the tissue with particular staining. The most common stainings are as follows: histological, histochemical, immunohistochemical, and immunofluorescence, used for different purposes [20]. In this work, we used virtual slides obtained by digital conversion of Hematoxylin and Eosin (H &E) stained glass slides, a histology stain widely used to analyze the nature of tissue sections under the microscope. Hematoxylin and Eosin (H&E) is a routine histopathology stain that allows highlighting of the nuclei of cells (with Hematoxylin) and the cell cytoplasm (with eosin).

One of the crucial issues of WSI employment in computer vision applications is the colossal image size. The most common technique to optimize the computational task is to tile them into small patches. WSI tiling can generate many patches, depending on the size and resolution of the virtual slide and the size of the patches. Tiling can be an automatic or handcrafted task. If automated, the patches can be randomly generated or led by a WSI analysis to find the most significant portions. Even if deep learning approaches perform very well in different computational pathology tasks, there are few comparisons among the different feature extraction architectures and few discussions about the relationships between machine and human vision attention.

In this work, we investigated the retrieval performance of features extracted from pre-trained Neural networks in PACSs. In particular, we compared different architectures chosen by their top-1 accuracy achieved on the validation split of ImageNet [21]. Moreover, we evaluated the features extracted from the top of the network and the middle layers to figure out how to work a general pre-trained CNN in the Computational Pathology domain. To the best of our knowledge, there are no studies in the literature about the most efficient way to extract the depth feature in the pathological field. For this reason, we deeply analyzed some CNNs architectures to find the most suitable way to represent features in this domain context and discuss the obtained results. Furthermore, we identified the best operations to reduce a three-dimensional feature map into a one-dimensional array for an efficient and effective CBIR task.

We organized the rest of the article as follows: the “Related Work” section introduces different related works related to our study; in the “Material and Methods” section, we present our proposed approach and methodology; the “Evaluation Strategy” section shows our evaluation strategy and reports the obtained results, the “Discussion” section is devoted to the discussion on results and, eventually, conclusion and future works are in the “Conclusion” section.

## Related Work

In this section, we analyzed different works on histopathology image retrieval, mainly focusing on feature representation. In [22], the authors presented an approach to represent histopathology knowledge for CBIR systems. It was accomplished by a semantic mapper based on SVM classifiers. This mapper allows for a new semantic feature space in which a metric measures the similarity between images. They used Gray histograms, Color histograms, Tamura texture histograms, and Sobel histograms and computed other meta-features on the histograms. The authors in [23] presented a framework to build histology image representations that combine visual and semantic features using the NMFA and NSA algorithms. Their method learns the relationships between both data modalities and uses that model to project semantic information back to the visual space building the fused representation. Ultimately, such representation is used in an image search system that matches potential results using a similarity measure. In the same way, in [24], the authors introduced an image retrieval framework for histopathological image analysis. Mainly, they focused on hashing-based retrieval methods and investigated a kernelized and supervised hashing approach for real-time image retrieval. Instead, in [25], the authors proposed a CBIR algorithm based on a hierarchical annular histogram (HAH) with a refinement schema based on dual-similarity relevance feedback. Jimenez et al. [26] proposed a multimodal case-based retrieval approach for histopathology cases based on visual features obtained with deep learning with an automatic description of pathology reports. Furthermore, they used a strategy fusing visual features from WSIs and text embeddings of pathology reports. The deep features representing WSIs, are generated with a CNN trained to classify cancer gradings. Moreover, authors in [27] proposed a complete size-scalable CBIR framework for a large-scale database of WSIs using the binarization method and hashing technique to feature identification and similarity measurement for the images represented in multiple binary codes. The primary operand of the proposed method is from the ranking step, which varies with the number of proposal regions. Also, in [28], authors produced compact features for image retrieval, reduced deep features, and deep barcodes derived from deep features of a pre-trained network. They used VGG16, VGG19, and AlexNet as deep feature extractors. In [29], a deep learning-based reverse image search tool for histopathology images is presented called Similar Medical Images Like Yours (SMILY). It allows pathologists to perform queries by example. They divided their application into two stages. The first one concerned the database creation from WSIs, tailing the images into patches, and extracting the depth features. The latter concerns the query process, where a patch is selected from query WSI, and the nearest neighbor search is performed. Likewise, in [30], authors proposed a digital pathology system with a WSI viewer to retrieve visually similar local areas in the same image and other images from an extensive database and open-access literature. The system evolves a DenseNet121 trained on breast cancer histopathological images and deep features extraction from patches. In [31], a retrieval and classification system for histological images based on local energetic information, local structural information, local geometric information, and local patterns of the textures using Riesz Transform and Monogenic local binary patterns (M-LBP). In [32], the authors use deep metric learning for the histopathological image retrieval task. They constructed the network with a mixed attention mechanism involving spatial and channel attention and trained with the multi-similarity loss under the supervision of category information. Yottixel [33] is an image search engine for digital pathology. It is based on a combination of supervised and unsupervised algorithms. In particular, it uses the VGG network, all Inception, DenseNet, and in-house trained CNNs. They used deep features to characterize patches extracted from WSIs.

In this article, we are interested in analyzing pre-trained CNN architectures to find the most suitable way to extract features and explain the most robust deep feature representations. That is a significant novelty compared with existing literature focused only on standard layer exclusion considering the last CNN layer. Moreover, we also highlight the importance of the feature maps dimensionality reduction, which is necessary to perform an efficient and effective retrieval task using vector-based algorithms in a comprehensive CBIR approach for PACS.

## Material and Methods

In this section, we introduced our methodology and technological choices to evaluate the performance of our CBIR system for the histopathological domain using features extracted from pre-trained CNNs. In this work, we focused on the patches obtained from WSIs because we would show the effectiveness of pre-trained CNNs as feature extractors highlighting their strengths and weaknesses.

**Fig. 1:**
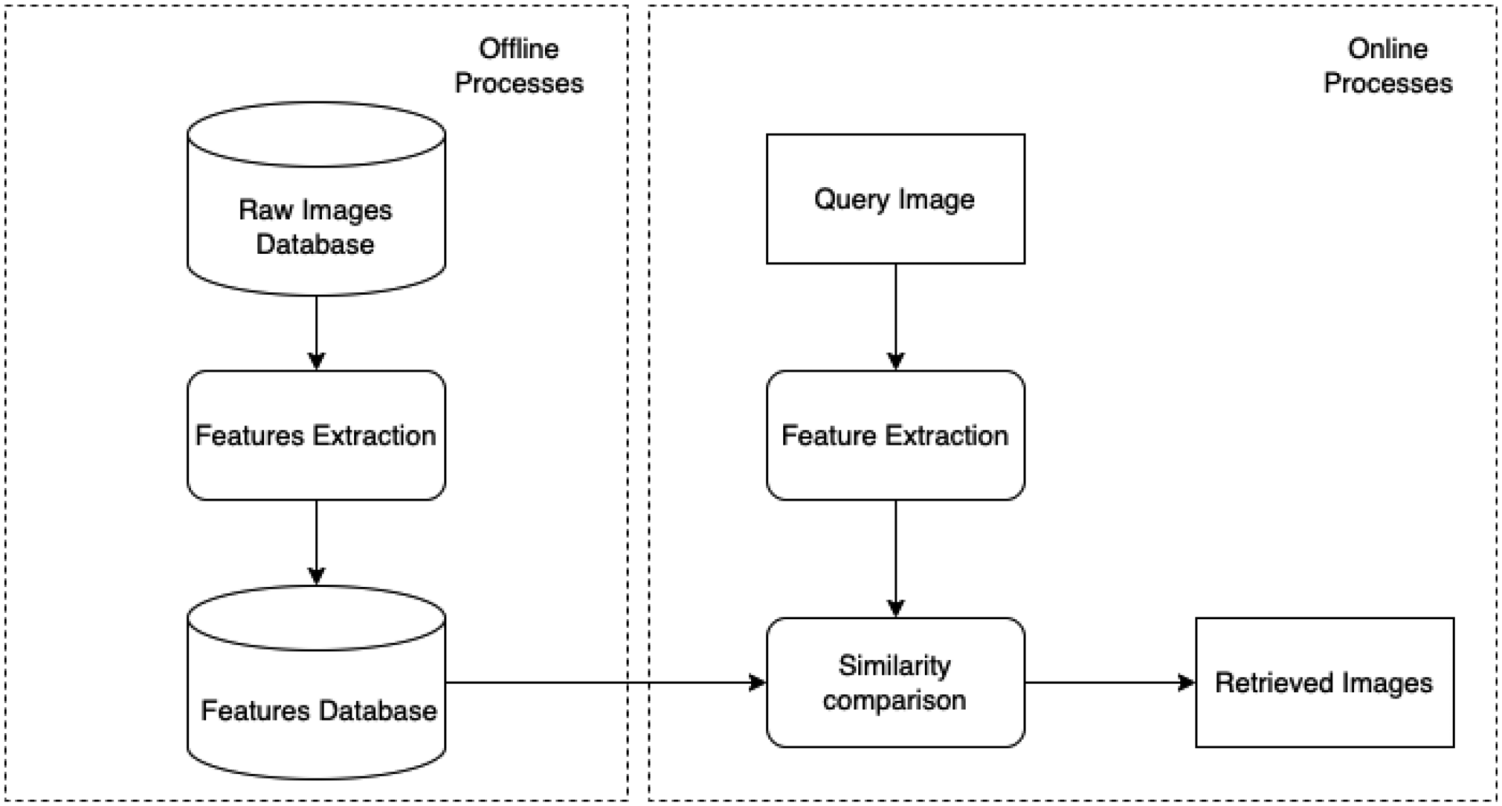
Content Based Image Retrieval (CBIR) architecture

Firstly, we presented our CBIR system depicted in Fig. 1. From a general point of view, we divided the workflow into two modules, the first one contains the offline processes, and the latter performs the online ones. The offline processes involve extraction and storing features from the H &E patches repository, and the online ones regard feature extraction from the query patch and comparing all stored features using a similarity function. To perform similarity, we represented each patch as a one-dimensional array and stored it with an associated label. The system extracts the one-dimensional array from the query patch at the time, computes the similarity using a distance measure between vectors, and ranks the results. The main issues are related to the chosen features and similarity functions. About the similarity function, in this work, we choose the cosine similarity, computed as one minus cosine distance due to its good performance [34]. Instead, for features, we choose to extract them from pre-trained CNNs. The first step is the selection of CNNs, and the second is the image preprocessing to arrange the CNNs input. In the following, we introduced the selected CNNs, the choice of the layers for each one, and the preprocessing operation performed on the dataset. According to [35], we choose the CNN for each kind of architecture that achieved the better top-5 accuracy on the validation split of ImageNet. Moreover, we accurately report the methodology used for the feature extraction step.

### Used Convolutional Neural Networks

Convolutional Neural Networks (CNNs) or Deep Convolutional Neural Networks (DCNNs) are a class of Artificial Neural Networks (ANNs) used for the analysis of visual contents. CNNs are mainly divided into two blocks. The first is responsible for feature learning, while the latter handles the classification process. Principal concepts applied in feature learning are convolution and pooling. The convolution involves some filter or kernel with trainable weights with the images to compute a feature map. Instead, pooling reduces the dimension of feature maps summarizing its patches using the mean of maximum operations. In the following, we briefly described the selected CNNs architectures and showed what layers we chose to extract the deep features. In the “Evaluation Strategy” section, we described our evaluation strategy to choose the best layer for each CNN, compared them, and discussed the precision of the related feature in the histopathological image retrieval task.

#### VGG-Net

VGG-Net [36] is a CNN architecture proposed by the Visual Geometric Group of Oxford University. The network uses convolutional layers with filters with very small receptive filed (3x3) with ReLU activations and 2x2 pooling max layers performed after some convolutional layers. The authors proposed two versions with sixteen and nineteen layers. In our framework, we choose VGG16, consisting of sixteen layers organized in five blocks, excluding the top. In particular, each block is composed of some convolutional layers followed by max-pooling layers. Furthermore, we considered depth features as the output of each block, applying global max/average pooling or flattened operation. Table 1 summarizes the selected layers with output shape and feature size after dimensional reduction where block_*i*_pool is the last layer of the *i*-th block.

**Table 1:** Deep feature size VGG16 for each selected layer

		Feature size	
Layer	Output shape	Max/Avg	flatten
block1_pool	112x112x64	64	802816
block2_pool	56x56x128	128	401408
block3_pool	28x28x256	256	200704
block4_pool	14x14x512	512	100352
block5_pool	7x7x512	512	25088

#### Inception V3

The Inception deep convolutional architecture was called GoogLeNet [37] and represents the Inception v1. Afterward, Inception v2 was introduced in [37], adding batch normalization. Later, in [38], Inception v3 was proposed with additional factorization concepts. In brief, the basic idea is factorizing convolution to reduce the number of connections and parameters without decreasing the network efficiency. This architecture employs four main kinds of modules: the first one (module 1) uses convolutional layers and implements small factorization convolutions; the second and third (modules 2 and 3) implement factorization into asymmetric convolutions; and the last one implements efficient grid size reduction. The final architecture consists of three Inception Module 1, one Grid Size Reduction Module, four Inception Module 2, one Grid Size Reduction Module, and two Inception Module 3. We choose as deep features the outputs of each module applying global max/average pooling or flattened operation. Table 2 summarizes the selected layers with output shape and feature size after dimensional reduction where mixed*i* is the output of the *i*-th module.

**Table 2:** Deep feature size Inception V3 for each selected layer

		Feature size	
Layer	Output shape	Max/Avg	flatten
mixed0	35x35x256	256	313600
mixed1	35x35x288	288	352800
mixed2	35x35x288	288	352800
mixed3	17x17x768	768	221952
mixed4	17x17x768	768	221952
mixed5	17x17x768	768	221952
mixed6	17x17x768	768	221952
mixed7	17x17x768	768	221952
mixed8	8x8x1280	1280	81920
mixed9	8x8x2048	2048	131072
mixed10	8x8x2048	2048	131072

#### Residual Network

Residual Networks (ResNet) [39] is an architecture of CNN designed to mitigate the vanishing gradient effect in deep networks. The main block is the residual block in its second version proposed in [40]. It consists of a stack of 1x1 - 3x3 - 1x1 convolutions layers where batch normalization and activation are applied before convolution. The version of ResNet differs by the number of layers. In this study, we choose ResNet152V2 which has 152 layers. The layers are grouped into four convolutional blocks composed of some residual blocks. We considered the output of each convolutional block as a deep feature applying global max/average pooling or flattened operation. Table 3 summarizes the selected layers with output shape, and feature size after dimensional reduction where conv_*i*_block*j*_out is the *j*-th output of the *i*-th module.

**Table 3:** Deep feature size ResNet152V2 for each selected layer

		Feature size	
Layer	Output shape	Max/Avg	flatten
conv2_block3_out	28x28x256	256	200704
conv3_block8_out	14x14x512	512	100352
conv4_block36_out	7x7x1024	1024	50176
conv5_block3_out	7x7x2048	2048	100352

#### Inception Residual Network

Inception-ResNet [41] was inspired by ResNet and Inception. There are two versions of this network architecture, namely v1 and v2. The architecture mainly employs six modules: Stem, Inception-resnet-A, Reduction-A, Inception-resnet-B, Reduction-B, and Inception-resnet-C. Inception-ResNet modules are similar to inception modules adding the residual connection. Reduction modules are like inception modules, and the Stem module performs convolutions and spatial pooling. The final network configuration in InceptionResNetV2 consists of one Stem Module, one Inception-A block, ten Inception-ResNet-A blocks, one Reduction-A block, twenty Inception-ResNet-B blocks, one Reduction-B block, ten Inception-ResNet-C blocks, and a final convolution block. We choose the output of the Inception-A block, Reduction-A block, Reduction-B block, and final convolution applying on each one global max/average pooling or flattened operation as deep features. Table 4 summarizes the selected layers with output shape and feature size after dimensional reduction where mixed_5b corresponds to 1, mixed_6a to 2, mixed_7a to 3, and conv_7b to 4.

**Table 4:** Deep feature size Inception-ResNetV2 for each selected layer

		Feature size	
Layer	Output shape	Max/Avg	flatten
mixed_5b	35x35x320	320	392000
mixed_6a	17x17x1088	1088	314432
mixed_7a	8x8x2080	2080	133120
conv_7b	8x8x1536	1536	98304

#### Xception

Xception (Extreme Inception) [42] was inspired by Inception V3 and ResNet. It mainly consists of three main flow entries, middle and exits repeated respectively one, eight, and one time. Each flow uses convolution with a receptive field of 3x3, spatial pooling, and separable convolution introduced in this architecture in inception-like. Furthermore, it has all residual connections. We selected as deep features the output of entry flow, the eight outputs of middle flow, and the output of exit flow, applying global max/average pooling or flattened operation to each considered layer. Table 5 summarizes the selected layers with output shape and feature size after dimensional reduction where add_2 corresponds to the output of entry flow, add_10 to the output of the last middle flow, and block_14_sepconv2 to the output of the exit flow.

**Table 5:** Deep feature size Xception for each selected layer

		Feature size	
Layer	Output shape	Max/Avg	flatten
add_2	19x19x728	728	262808
add_10	19x19x728	728	262808
block14_sepconv2	10x10x2048	2048	204800

#### Dense Convolutional Network

Dense Convolutional Network (DenseNet) [43] introduced a direct connection between any two layers with the same feature fmap size. DenseNet mainly employs two components: dense block and transition layers. The architecture switch between them to build a deep network. In a dense block, each layer receives collective knowledge from preceding layers. Practically, each layer receives additional input from all preceding layers and gives its feature maps to all following layers. A transition layer controls the complexity of the model, reducing the number of channels by using 1x1 convolutional layers and halving the height and width of the average pooling layer. In our study, we choose DenseNet201, that have two hundred-one layers, considering the output of each transition layer and the last dense block after global average/max pooling or flatten operation as deep features. Table 6 summarizes the selected layers with output shape and feature size after dimensional reduction where pool_*i* corresponds to output *i*-th transaction layer, and con5_block32_concat is the output of the last dense block.

**Table 6:** Deep feature size DenseNet201 for each selected layer

		Feature size	
Layer	Output shape	Max/Avg	flatten
pool2_pool	28x28x128	128	100352
pool3_pool	14x14x256	256	50176
pool4_pool	7x7x896	896	43904
conv5_block32_concat	7x7x1920	1920	94080

#### NASNet

The authors in [44] proposed an architectural block of CNN using a deep reinforcement learning method. They specified the general architecture arranged as some normal cells followed by a reduction cell. They used a Recurrent Neural Network (RNN) to predict some characteristics of the network as the number of normal cells and the architecture of cells. A normal cell is a convolutional block that gives back a feature map of the same dimension, while a reduction cell is a convolutional block that gives back a feature map where the feature map height and width are reduced by a factor of two. They trained the first version of CNN on the CAFIR dataset and adapted it on ImageNet. We chose NasNetLarge, a version trained on ImageNet, and we considered the output of each block of normal cells and reduction cells as a deep feature after applying global max/average pooling or flatten operation. Table 7 summarizes the selected layers with output shape and feature size after dimensional reduction, where layers normal_concat_5, normal_concat_12 and normal_concat_12 are the last layers of a series of normal cells, while normal_concat_reduce_6 and normal_concat_reduce_12 are the last layers of reduction cells.

**Table 7:** Deep feature size NASNetLarge for each selected layer

		Feature size	
Layer	Output shape	Max/Avg	flatten
normal_concat_5	42x42x1008	1008	1778112
reduction_concat_reduce_6	21x21x1344	1344	592704
normal_concat_12	21x21x2016	2016	889056
reduction_concat_reduce_12	11x11x2688	2688	325248
normal_concat_18	11x11x4032	4032	487872

#### MobileNet

MobileNet [45] is a CNN architecture inspired by InceptionNet to work on mobile devices, with the primary goal of reducing the number of parameters and computations and preserving the performance as much as possible. The authors introduced a filter architecture called Depthwise Separable Convolution that split the computation into two steps: (i) it applies a single convolutional filter for each input channel, and (ii) it uses pointwise convolution to create a linear combination of the output. In our study, we chose MobileNetV2 [46], an improvement of MobileNet in which the authors added an inverted residual connection and highlighted the importance of linear bottlenecks. This version has two main blocks, one with and one without residual connection. The final architecture consists of sixteen blocks. We considered the output of each block and the last one excluding dense layers as a deep feature after applying global max/average pooling or flatten operation. Table 8 summarizes selected layers with output shape and feature size after dimensional reduction, where block_*i*_project_BN is the last layer of a *i*-th block without residual connection, block_*i*_add is the last layer of *i*-th block with residual connection, and out_relu is the last layer for feature extraction.

**Table 8:** Deep feature size MobileNetV2 for each selected layer

		Feature size	
Layer	Output shape	Max/Avg	flatten
block_1_project_BN	56x56x24	24	75264
block_2_add	56x56x24	24	75264
block_3_project_BN	28x28x32	32	25088
block_4_add	28x28x32	32	25088
block_5_add	28x28x32	32	25088
block_6_project_BN	14x14x64	64	12544
block_7_add	14x14x64	64	12544
block_8_add	14x14x64	64	12544
block_9_add	14x14x64	64	12544
block_10_project_BN	14x14x96	96	18816
block_11_add	14x14x96	96	18816
block_12_add	14x14x96	96	18816
block_13_project_BN	7x7x160	160	7840
block_14_add	7x7x160	160	7840
block_15_add	7x7x160	160	7840
block_16_project_BN	7x7x320	320	15680
out_relu	7x7x1280	1280	62720

#### EfficientNet

EfficientNet [47] is a family of models that are optimized to have few parameters and be faster. This model is scalable in depth, width, and resolution. The authors developed a baseline network using a multi-objective NAS [48]. The main layers are Mobile inverted Bottleneck Convolution (MBConv) and Squeeze Excite (SE). EfficientNetV2 [49] has been improved using progressive learning and replacing some MBConv layers with Fused-MB Conv [50]. It uses NAS to search for the best combination of fused and regular MB Conv Layers. In our work, we used EfficentNetV2L, where L stands for large. This architecture is scaled up in depth, width, and resolution. It consists of seven wider blocks for feature learning, followed by batch normalization, activation, and top layers. We chose the output of each block and the last one excluding dense layers as an in-depth feature after applying global max/average pooling or flatten operation. Table 9 summarizes the selected layers with output shape and feature size after dimensional reduction, where block*ij*_add is the output of *i*-th block, *j* stands for the last sub-blocks that envelope it in width, and top_rule is the last layers excluding top classification layers.

**Table 9:** Deep feature size EfficientNetV2L for each selected layer

		Feature size	
Layer	Output shape	Max/Avg	flatten
block1d_add	240x240x32	32	1843200
block2g_add	120x120x64	64	921600
block3g_add	60x60x96	96	345600
block4j_add	30x30x192	192	172800
block5s_add	30x30x224	224	201600
block6y_add	15x15x384	384	86400
block7g_add	15x15x640	640	144000
top_activation	15x15x1280	1280	288000

### Preprocessing

The images given in input to Convolution Neural Networks, especially for a pre-trained one, must be processed to have the correct representation according to the training format. In particular, the input must be equal to the one used in the training step, and each pixel value must be normalized according to the used architecture. In particular, each input has three channels because images are RGB, and it must be 224x224 for VGG16, ResNet152V2, MobileNetV2, and DenseNet201, 229x299 for InceptionV3, InceptionResNetV2 and Xception, 331x331 for NASNetLarge, and 480x480 for EfficientNetV2. Table 10 summarizes the input shape and the operation computed by the preprocessing pipeline.

**Table 10:** Input size and preprocessing input function for each CNN

CNN	Input Shape	Preprocess Input Function
VGG16 [36]	224x224	It converts RGB to BGR, The images are converted from RGB to BGR, then each color channel is zero-centered with respect to the ImageNet dataset, without scaling.
InceptionV3 [38]	299x299	The inputs pixel values are scaled between -1 and 1, sample-wise.
ResNet152V2 [40]	224x224	The inputs pixel values are scaled between -1 and 1, sample-wise.
InceptionResNetV2 [41]	299x299	The inputs pixel values are scaled between -1 and 1, sample-wise.
MobileNetV2 [46]	224x224	The inputs pixel values are scaled between -1 and 1, sample-wise.
DenseNet201 [43]	224x224	The input pixels values are scaled between 0 and 1 and each channel is normalized with respect to the ImageNet dataset.
Xception [42]	299x299	The inputs pixel values are scaled between -1 and 1, sample-wise.
NasNetLarge [44]	331x331	The inputs pixel values are scaled between -1 and 1, sample-wise.
EfficientNetV2L [49]	480x480	Nothing

**Fig. 2:**
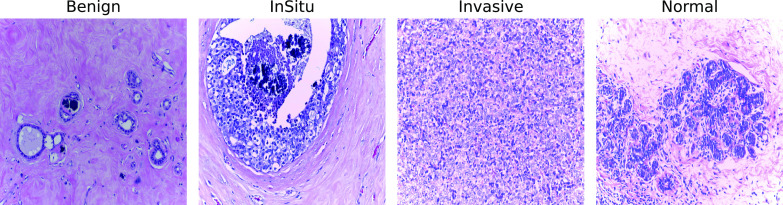
Example patches from ICIAR 2018 Grand Challenge on Breast Cancer Histology (BACH). From right to left, the first image depicts a benign tumor example, the second an in situ tumor, the third an invasive tumor, and the last one does not contain a tumor

### Dataset

In this work, we use a standard dataset called BACH, provided by ICIAR 2018 Grand Challenge on Breast Cancer Histology [51]. This dataset contains Hematoxylin and Eosin (H &E) staining breast histology microscopy whole-slide images. Furthermore, it also has the patches extracted from WSI, and due to our intent to identify the best deep feature for a CBIR system, we used it. The dataset contains 400 images divided into four classes: (i) invasive, (ii) in situ, (iii) benign, and (iv) normal. Figure 2 shows an example for each class applying stain color normalization.

## Evaluation Strategy

This section presents and discusses our experimental results and their evaluation. We conducted several experiments to identify the best CNN, particularly its best-performing layer with related dimensionality reduction techniques. The computational system architecture used to run experiments is:

- CPU: Intel(R) Core(TM) i7-4790K CPU @ 4.00GHz
- RAM: 32GB DDR3
- GPU: NVIDIA GeForce RTX 3090 24 GB
- Operation System (OS): Ubuntu Server 20.04.4 LTS

We are not interested in the task execution speed time at this research stage.

On the other hand, we pay attention to the measure of system accuracy in the retrieval task. Different metrics such as precision, precision at k, recall, f-measure, precision-recall curve, and Mean Average Precision, mean Average Precision at K are presented in literature [34] and, we used precision at k (P@k) because we are interested in the first k relevant results. In detail, as reported in Eq. 1, P@k calculates how many retrieved items on K top-ranked ones are relevant for a given query. We computed the P@k on four values of K: 5, 10, 50, and 100.

1 $$\begin{aligned} P@k=I_r/K \end{aligned}$$

Moreover, we also consider the average of P@K (MAP@k) to evaluate the results from all queries. Furthermore, we used a confusion matrix to analyze the results and understand how the CBIR task works for each category available in the used dataset. In particular, rows of the confusion matrix contain the query category, and the columns are the category of retrieved images. Each value concerns precision at k of *j*-th category retrieved for the *i*-th category queried, *i* is the index of the row, and *j* of the column. We used each dataset image as a query by example, obtaining for each experiment 400 queries. The image used as the query is left out from the results set. In order to perform a robust evaluation, we focused on analyzing three crucial aspects to justify the choice made by quantifying the loss/gain of the dimensionality reduction method, the layer selection for each CNN, and, ultimately, the CNNs architecture.

### Results

As previously stated, we conducted three analyses to understand our results better. Firstly, we analyzed all results identified by each CNN and considered layer and reduction operations to find the best way to reduce the feature map from a 3-D to a 1-D array. On the other hand, we set the reduction operation and analyzed the result to recognize the best layer for each CNN to extract the deep features. Eventually, we compared all CNNs to find the best result. We quantified the loss/gain in terms of P@k in each analysis.

**Table 11:** Average Gain/Loss P@k quantification by global average pooling for each CNN

CNN	Reduction	Avg-GLP@5	Avg-GLP@10	Avg-GLP@50	Avg-GLP@100
DenseNet201	flatten	0.1555	0.1281	0.0597	0.0428
	max	0.0526	0.0431	0.0091	0.0066
EfficientNetV2L	flatten	0.1905	0.1381	0.0560	0.0338
	max	0.1978	0.1571	0.0852	0.0568
InceptionResNetV2	flatten	0.1395	0.1018	0.0438	0.0311
	max	0.0675	0.0419	0.0142	0.0091
InceptionV3	flatten	0.2195	0.1810	0.0936	0.0639
	max	0.0790	0.0617	0.0273	0.0195
MobileNetV2	flatten	0.1566	0.1195	0.0509	0.0333
	max	0.1172	0.0873	0.0345	0.0210
NASNetLarge	flatten	0.1960	0.1639	0.0904	0.0608
	max	0.0902	0.0716	0.0367	0.0233
ResNet152V2	flatten	0.1816	0.1464	0.0685	0.0435
	max	0.0359	0.0248	**-0.0052**	**-0.0081**
VGG16	flatten	0.1886	0.1487	0.0671	0.0425
	max	0.0661	0.0452	0.0107	0.0046
Xception	flatten	0.1718	0.1393	0.0734	0.0530
	max	0.0462	0.0307	0.0105	0.0068

In bold the best results

#### Dimensionality Reduction Methods

We computed P@5, P@10, P@50, and P@100 for each CNN layer and reduction operation to identify the best dimensionality reduction method. Our experiments show that the global average pooling obtains, on average, the best results on each layer at each precision. To quantify the improvement of global average pooling rather than global max pooling or flattening, we defined the loss/gain precision at k (GLP@k) for each precision level and reduction method as reported in Eq. 2. It computes the difference between P@k for global average pooling and P@k for flattening, where *m* is the reduction method for which we want to measure the gain or loss of precision of k concerning the global average pooling.

2 $$\begin{aligned} GLP_m@k = P_{avg}@k - P_m@k \end{aligned}$$

Table 11 summarizes the GLP@k for each CNN, where we calculated each value as the average of GLP@k on each layer. A positive value means that global average pooling has a gain. Otherwise, it has a loss. Results show that the global average pooling is the most suitable for *k* equal to 5 and 10, while for *k* equal to 50 and 100, we have a slight loss for ResNet152V2, there is a slight loss. Therefore, from a global point of view and our interest in having a high precision in the first k results, the global average pooling is the best choice to maximize the precision at each level.

**Table 12:** The best CNNs layer for each precision level

CNN	Best Layer (P@5)	Best Layer (P@10)	Best Layer (P@50)	Best Layer (P@100)
DenseNet201	conv5_block32_concat (0.67)	conv5_block32_concat (0.626)	conv5_block32_concat (0.4839)	conv5_block32_concat (0.4123)
EfficientNetV2L	block6y_add (0.7365)	block6y_add (0.671)	block6y_add (0.4998)	block6y_add (0.4192)
InceptionResNetV2	mixed_7a (0.68)	mixed_7a (0.6325)	mixed_7a (0.495)	mixed_7a (0.4177)
InceptionV3	mixed6 (0.693)	mixed6 (0.6298)	mixed6 (0.4864)	mixed6 (0.411)
MobileNetV2	block_13_project_BN (0.6655)	block_13_project_BN (0.604)	block_14_add (0.471)	out_relu (0.4025)
NASNetLarge	Normalizational_concat_12 (0.695)	Normalizational_concat_12 (0.6295)	Normalizational_concat_12 (0.4988)	reduction_concat_reduce_12 (0.4197)
ResNet152V2	conv4_block36_out (0.6475)	conv4_block36_out (0.5923)	conv4_block36_out (0.4586)	conv4_block36_out (0.3902)
VGG16	block4_pool (0.6125)	block5_pool (0.562)	block5_pool (0.4532)	block5_pool (0.3871)
Xception	add_10 (0.671)	add_10 (0.6185)	add_10 (0.4771)	add_10 (0.4051)

**Table 13:** Gain/Loss Precision at k for CNN where the best layer is not equal for each k

CNN	Layer	Layer1	P@5	P@10	P@50	P@100
MobileNetV2	block_14_add	block_13_project_BN	**-0.0050**	**-0.00400**	0.01255	0.007475
		out_relu	0.0270	0.00850	0.00140	**-0.003925**
NASNetLarge	Normalizational_concat_12	reduction_concat_reduce_12	0.0295	0.00900	0.00535	**-0.000275**
VGG16	block5_pool	block4_pool	**-0.0130**	0.01575	0.03430	0.024600

In bold the best results

#### Layer Selection for Each CNN

To find the best layers for each CNN, we computed the P@k for each layer, and if the best one was the same for each level of precision, we chose it. Otherwise, we estimated the loss gain P@k to find the best layer, which minimizes the loss.

As summarized in Table 12, for DenseNet, EfficientNetV2, InceptionResNetV2, InceptionV3, ResNet152V2 and Xception, the best layers are respectively conv5_block32_concat, block6y_add, mixed_7a, mixed6, conv4_block36_out and add_10 at each level of precision; for MobileNetV2, the best layer is block_13_project_BN for precision equal to 5 and 10, block14_add for precision equal to 50, and out_relu at precision equal to 100; for NasNetLarge, the best layer is normal_concat_12 for precision equal to 5, 10 and 50, and reduction_concat_reduce_12 for precision equal to 100; for VGG16, the best layer is block4_pool for precision equal to 5 and block5_pool for precision equal to 10, 50 and 100. Table 13 shows the gain/loss precision at k for MobileNet, NasNetLarge, and VGG16.

According to the results, we chose block_14_add for MobileNetV2 because it outperforms out_relu and has a slight gain on block_13_project_BN. We chose normal_concat_12 for NASNetLarge and block5_pool for VGG16 because they have better results than the other layers.

#### Best Convolutional Neural Network Recognition

**Fig. 3:**
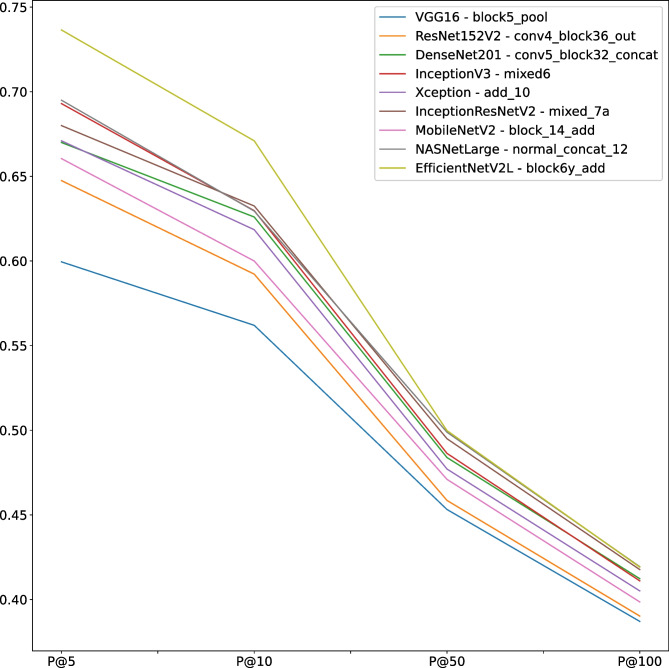
Precision comparison at 5, 10, 50 and 100 obtained from retrieval using CNN layers set according to results reported in the “Results” section and global average pooling to reduce dimensionality

**Fig. 4:**
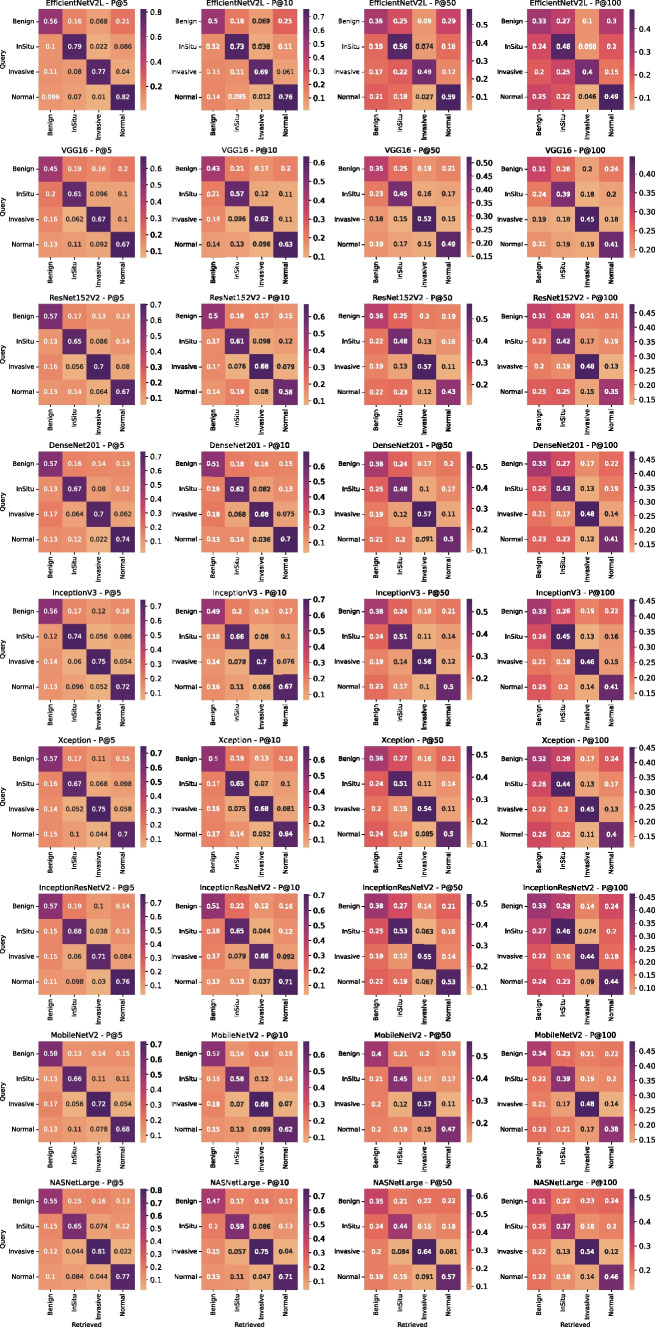
Comparison of confusion matrices obtained from retrieval using CNN layers set according to results reported in the “Results” section and global average pooling to reduce dimensionality

To find the best CNN to use as a feature extractor, we computed the P@k for each one fixing the reduction method with global average pooling and the best layer according to the ones above recognized. Table 14 shows the results for each configuration, highlighting that the best CNN is EfficientNetV2. It is best for k equal to 5 and 10, but for k equal to 50 and 100, the gap with other CNN is slight, as displayed in Fig. 3. We remark that we are interested in high precision on top k results. To accomplish a more precise analysis, we analyzed in depth all the best CNN configurations using a confusion matrix. We intend to understand which dataset category is not correctly retrieved. According to Fig. 4, it is clear that the queries using benign examples are not correctly retrieved. They are often misunderstood with in situ images and normal ones. Furthermore, normal and invasive queries are usually recognized with good precision value. In particular, all networks have good performance for k equal to 5 and 10 but the performance degrading for k equal to 50 and 100, especially for benign queries. In the next section, we will give a qualitative explanation analyzing the worst and better results.

**Table 14:** Average P@k for each chosen layer

model	Layer	P@5	P@10	P@50	P@100
VGG16	block5_pool	0.5995	0.56200	0.45320	0.387075
ResNet152V2	conv4_block36_out	0.6475	0.59225	0.45855	0.390225
DenseNet201	conv5_block32_concat	0.6700	0.62600	0.48390	0.412325
InceptionV3	mixed6	0.6930	0.62975	0.48635	0.411025
Xception	add_10	0.6710	0.61850	0.47710	0.405100
InceptionResNetV2	mixed_7a	0.6800	0.63250	0.49500	0.417700
MobileNetV2	block_14_add	0.6605	0.60000	0.47100	0.398575
NASNetLarge	Normalizational_concat_12	0.6950	0.62950	0.49885	0.419425
**EfficientNetV2L**	**block6y_add**	**0.7365**	0.**67100**	**0.49980**	**0.419150**

In bold the best results

## Discussion

**Fig. 5:**
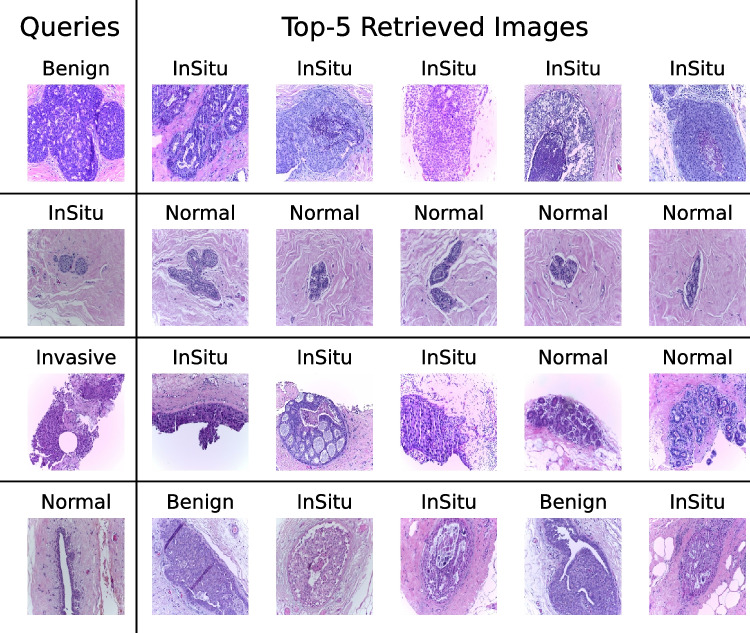
Examples of the wrong retrieval, in all cases, P@5 is equal to 0

**Fig. 6:**
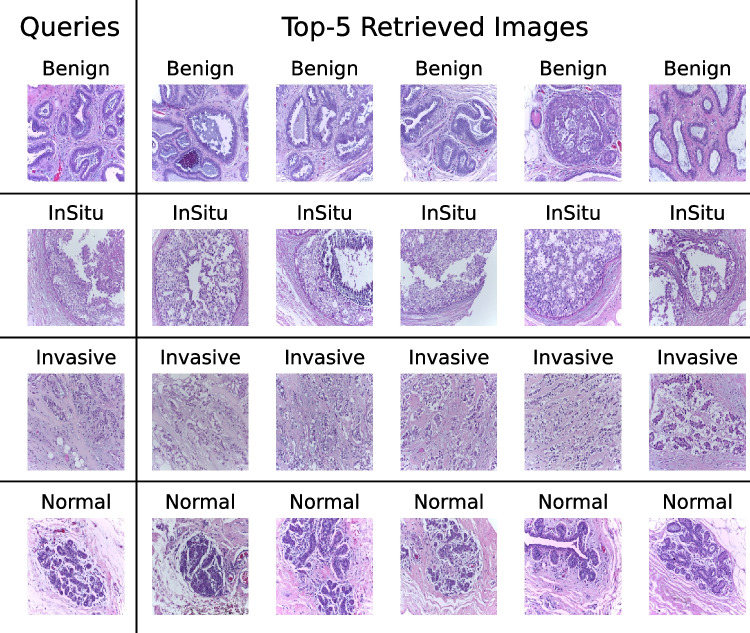
Examples of the correct retrieval, in all case, P@5 is equal to 1

In this section, we provide a qualitative analysis of the worst results to understand why the retrieved images are not correct for some queries. The morphological patterns associated with breast disease at histopathology examination can be highly heterogeneous. Therefore, the diagnostic assessment considers all the morphological patterns recognized by the pathologist, at the microscope, during the histopathology evaluation. The pathologist reports all the characteristics observed at the histopathological examination, noting them in the report. Due to the heterogeneity mentioned above, the annotation produced to create the ground truth of a breast dataset could be oversimplified. In other words, the unsupervised and random production of patches from a WSI might occasionally provide pictures that only represent a small region and can partially represent morphological patterns other than the ground truth. This is the case, for instance, of the “benig” query in Fig. 5, which the model may misclassify since it can be partially superimposed on an in situ framework in the image represented by the retrieved patches. In the mentioned case, although the patch comes from a “Benign” classified case, the image refers to a borderline morphological pattern that, in our opinion, could pose a differential diagnosis issue usually ruled out by immunohistochemistry, looking for p63 expression. The same might be stated for “normal” images retrieved in response to the “in situ” query Fig. 6. Although belonging to WSI annotated as “in situ”, the patch seems to refer more to a normal pattern. The remaining misclassifications could also potentially be explained by the bias of the patch’s field. In the case of the “invasive” query, there could be a bias related to the type of sample, which does not seem to be a whole section but a more undersized biopsy showing a not-so-clear morphology pattern (see Fig. 5).

## Conclusion

This work explored the feature representation of Hematoxylin and Eosin patches for CBIR tasks in digital pathology PACSs. Our results showed that not all analyzed CNNs had the best results at the last layers. For example, EfficinetNetV2 reached the best precision on block6y_add, which is not the last layer. Moreover, we empirically demonstrated that the global average pooling is the best way to reduce the dimension of a three-dimensional array into a one-dimensional one. Furthermore, we did a qualitative analysis of obtained results to understand why the precision is low in some cases. The qualitative analysis showed that the morphology overlapped in many incorrectly categorized instances, mainly due to the tiny patches’ low representativeness of the full WSI. Given the complexity of mammary gland histopathology, we will use other datasets to better analyze the performance of our approach together with WSI to test our framework in a real scenario. Moreover, we will set up a more precisely annotated dataset to reduce the misclassification rate due to an un-precisely patched annotation. Image retrieval algorithms are highly topical in the field of digital pathology. The number of images a pathology service can generate daily is around hundreds of units for a medium-sized service. Digital content, as in every other field, accumulates increasingly. Therefore, the need to find effective data recovery systems is growing to make the most of digital resources for clinical, scientific, and educational purposes. In future work, we will explore the effect of stain color normalization, which is often used to improve precision in a query-by-example approach. Furthermore, we will explore other levels of CNN to understand if others and not only the main layers can improve the results. Again, we will apply this approach to other datasets with characteristics like BACH to verify that results can be reproducible on other images. Further, we will investigate the use of these techniques on data that contains other tissue and tumor kind to understand if it is possible to achieve the same results independently from the slides’ origin.

## Data Availability

The dataset used in this work is public and correctly cited in the paper.
